# Differential Patterns in Motivations for Practicing Sport and Their Effects on Physical Activity Engagement across the Lifespan

**DOI:** 10.3390/healthcare11020274

**Published:** 2023-01-16

**Authors:** Marcelo de Maio Nascimento, Élvio Rúbio Gouveia, Bruna R. Gouveia, Adilson Marques, Cíntia França, Pedro Campos, Francisco Martins, Jesús García-Mayor, Andreas Ihle

**Affiliations:** 1 Department of Physical Education, Federal University of Vale do São Francisco, Petrolina 56304-917, Brazil; 2 Department of Physical Education and Sport, University of Madeira, 9020-105 Funchal, Portugal; 3 LARSYS, Interactive Technologies Institute, 9020-105 Funchal, Portugal; 4 Center for the Interdisciplinary Study of Gerontology and Vulnerability, University of Geneva, 1205 Geneva, Switzerland; 5 Regional Directorate of Health, Secretary of Health of the Autonomous Region of Madeira, 9004-515 Funchal, Portugal; 6 Saint Joseph of Cluny Higher School of Nursing, 9050-535 Funchal, Portugal; 7 CIPER, Faculty of Human Kinetics, University of Lisbon, 1495-751 Lisbon, Portugal; 8 ISAMB, Faculty of Medicine, University of Lisbon, 1649-020 Lisbon, Portugal; 9 Department of Informatics Engineering and Interactive Media Design, University of Madeira, 9020-105 Funchal, Portugal; 10 Public Health and Epidemiology Research Group, San Javier Campus, University of Murcia, 30720 San Javier, Spain; 11 Department of Psychology, University of Geneva, 1205 Geneva, Switzerland; 12 Swiss National Centre of Competence in Research LIVES—Overcoming Vulnerability: Life Course Perspectives, 1015 Lausanne, Switzerland

**Keywords:** physical activity, sport, motivator, lifespan, aging, vulnerability

## Abstract

This study aims to report what motivates individuals to be physically active, to determine whether motivating factors influence physical activity (PA) levels, and whether this differs across the lifespan. This is a cross-sectional study with 498 individuals: 117 adolescents, 306 adults, and 75 older adults. PA was assessed using Baecke’s questionnaire, and motivating factors for practicing sports were investigated using a scale with twelve questions. The factor analysis identified three motivating factors for sports practice: psychosocial, bodily, and well-being. The scale’s overall reliability and internal consistency indicated a Cronbach’s alpha of 0.885. The analysis of covariance (ANCOVA) adjusted for sex indicated the three factors as having a significant effect on PA (*p* < 0.050); however, only the well-being factor showed a significant interaction with age groups (*p* = 0.023, *η_p_*^2^ = 0.030). Subsequently, the effect of the well-being factor on PA scores in each age group was explored through regression analyses. Only older adults showed a significant association in the unadjusted [OR = 0.378, *p* = 0.001, R^2^ = 0.243] and the sex-adjusted analysis [OR = 0.377, *p* = 0.001, R^2^ = 0.288]. These results help us to better understand the underlying motivational reasons in different age groups for engaging in sports.

## 1. Introduction

Physical activity (PA) is considered one of the most effective strategies for combatting several factors associated with age-related vulnerability, such as health conditions [1,2]. It is a fact that an individual’s involvement in PA as well as a reduction in sedentary time are associated with improved mental, cognitive, and physical health [3]. However, progress worldwide in increasing levels of PA in populations is not at a sufficient rate [4]. In 2018, a population-based study reported that the global prevalence of age-standardized insufficient physical activity in the year 2016 was 27.5% [5]. Thus, the prediction was that, if this trend continued, it would not be possible to achieve the global PA target in 2025.

According to the World Health Organization (WHO) [4], more than 80% of the world’s adolescent population is insufficiently physically active. To reach adequate levels of PA, they must practice daily at least 60 min of aerobic exercise of moderate–vigorous intensity [6,7]. In addition, it is recommended that muscles and bones be strengthened at least three days a week. Moreover, teens must limit their daily sedentary time, particularly recreational screen time. The WHO recommends that individuals aged 18–65 years or older practice at least 150 to 300 min of aerobic PA at a moderate intensity or 75 to 150 min of aerobic PA at a vigorous intensity, or even the equivalent combination of aerobic PA at moderate and vigorous intensities [8]. In addition to aerobic training, muscle strengthening two or more days a week is recommended. It is important for the older adult population to include additional balance exercises three or more times a week.

However, voluntary participation/adherence to sports activities is a dynamic and complex process interdependent with several factors [9]. In this sense, the literature has highlighted factors that motivate individuals to practice PA. Among them, there is the desire to improve physical health (i.e., improvement of muscle, heart, and lung condition, decrease in bodily pain, and weight loss and control) [10]. Another motivating factor is the mental and psychological benefits (i.e., the opportunity to experience moments of pleasure, achieve stress relief, and feel good) [11,12]. A third motivational class for increasing PA levels is associated with the social benefits that sports practice offers (i.e., social contact and the opportunity to make and maintain friendships) [12,13]. Moreover, it is known that throughout life and depending on the age group, a series of factors (i.e., interpersonal, environmental, and individual) can vary and influence PA [14], motivating or discouraging an individual to practice exercises.

In this context, barriers to the practice of physical exercises arise, which in turn are decisive for an individual to present or not present adequate levels of PA [14,15]. The literature has reported a series of impediments to the practice of physical exercise, such as presenting health problems [9], not having a companion to go to training with [12], lack of time [10], or even lack of financial resources [14]. In addition, there are barriers related to the environment [16], such as transportation problems [17] and places with reduced accessibility [12], which in turn generate the fear of falling [10]. All these barriers contribute to a sedentary lifestyle, which in turn can accelerate the onset of diseases [18], and consequently increase the chances that individuals will become fragile and vulnerable to a series of life circumstances [19], consequently affecting their autonomy and adaptation to the environment [20].

Thus, the number of barriers to increasing PA levels may exceed the number of motivating factors, which presents a threat to the consolidation of healthy aging [4]. On the other hand, when individuals feel motivated to practice sports, the chances for an increase in PA levels become real [1,2]. In a population-based study carried out in the Portuguese population (15 to 84 years old) between 2015 and 2016, it was found that the prevalence of PA was decreasing with age [21]. Adolescents and young adults (15 to 21 years old) were the age group with the best PA levels (36%), followed by adults (27%) and older adults (22%). In the Autonomous Region of Madeira, where this study was carried out, there was a prevalence of 36.4% for sedentary behavior. Thus, in recent years, the National Health Plan and the National Program for the Promotion of Physical Activity have been creating policies across the country that make it possible to reduce sedentary behavior [22]. Measures include education campaigns for a better understanding of physical health, as well as strategies to motivate the Portuguese population to adhere to healthy lifestyle habits.

Over the years, review studies have reported barriers to and/or motivating factors for promoting PA levels in different age groups [16,23,24]. However, there is a lack of studies, mainly in the Portuguese population, that deepen the understanding of the different motivational patterns for sports and their effects on PA in different age groups throughout life (i.e., adolescence, adulthood, and older adulthood). Thus, to address these important gaps in the previous literature, this study aims to (1) report what motivates individuals in different age groups across the lifespan to be physically active and (2) determine whether those motivating factors influence total PA levels and whether this differs across the lifespan. Based on the previous literature, it was hypothesized that (1) according to age group (i.e., adolescents, adults, and older adults), there may be differences between motivating factors [25], while the exact pattern will have to be specified, and (2) compared with adolescents and adults, older adult engagement in sports may be more strongly motivated by the benefits of physical and mental health [10,11].

## 2. Materials and Methods

### 2.1. Participants

This cross-sectional study included 498 individuals residing in the Autonomous Region of Madeira, Funchal, Portugal. Study participants were grouped into 3 groups according to their ages: adolescents 12 to 17 years old (*n* = 117), adults 18 to 59 years old (*n* = 306), and older adults 60 to 89 years old (*n* = 75). All participants were part of the project entitled “Movement and Health: A Study on Sports Practitioners for All in the Autonomous Region of Madeira” (MOVeS). Recruitment took place in clubs, academies, cultural and sports centers, and sports associations between January and August 2017. The following inclusion criteria were considered: (1) being affiliated with a sports association, club, or other organization that promotes activities of PA, and (2) practicing any PA regularly. The exclusion criteria were as follows: (1) any medical contraindications for submaximal exercise according to the American College of Sports Medicine guidelines [26]; (2) inability to understand the study evaluation protocol; and (3) those who did not have individual medical insurance from the association/organization/sports club to practice PA did not participate for legal reasons. This study was scientifically and ethically approved by the Scientific Committee of the Department of Physical Education and Sport of the University of Madeira (reference: ACTA n.º 84; January 17, 2017) and by the Regional Secretariat for Education and Culture. Before participating in the evaluations, all members read and signed an informed consent form. The present study adhered to the Declaration of Helsinki. The evaluations were carried out by a team of investigators with experience and previously qualified for the application of the protocols. All procedures were performed at the Laboratory of Human Physical Growth and Motor Development at the UMa.

### 2.2. Measures

#### 2.2.1. Demographic Data

Demographic information was collected through face-to-face interviews based on a questionnaire. For the present study, the following information was asked: age, sex, and highest educational level reached, which was classified as (1) no schooling, (2) 1st cycle, (3) 2nd cycle, (4) 3rd cycle, (5) high school level, (6) bachelor’s degree, (7) master’s degree, or (8) doctorate.

#### 2.2.2. Physical Activity

Through face-to-face interviews, PA was assessed using the Baecke questionnaire [27]. For its validation, see Gouveia et al. [28]. This questionnaire allows the investigation of PA levels relative to the last 12 months. The questions comprise the following domains: (1) work/domestic work (PA-work); (2) sports activities (PA-sport)—regular activities lasting at least one hour per week; and (3) free time activities (PA-leisure). In the present study, the total PA score (PA-total) was calculated with the equation PA-total = PA-work + PA-sport + PA-leisure/3.

#### 2.2.3. Motivations for Practicing Sports

The evaluation of the motivating factors for the practice of sports was carried out using an instrument developed by the field team of the present study. The procedures included different phases (i.e., research, selection, creation, pilot/test, application, and reformulation). The questionnaire was based on review studies [29] and a meta-analysis [23] focusing on motivation to practice sports and/or participation in physical activities. Moreover, the questions were adapted from instruments applied by previous studies in individuals of different age groups [30,31,32]. Finally, a measurement instrument was obtained with simple language and accessibility for all age groups (i.e., adolescents, adults, and older adults), in addition to maintaining the evaluative objectives proposed by the original versions. The twelve questions were as follows: (1) be in shape; (2) to feel good; (3) be in good health; (4) maintain an adequate weight; (5) improve my appearance; (6) increase self-esteem and self-confidence; (7) produce positive psychological effects; (8) for fun; (9) reduce my stress levels and relax; (10) help reduce the pressure of everyday life; (11) make friends; and (12) lose weight. A five-level scale was presented for the answers, which ranged from 1 (strongly disagree) to 5 (strongly agree). The evaluation followed two different strategies: (1) sum of the total scores of the twelve questions (continuous variable) and (2) use of a factor analysis to construct factors in order to summarize the set of the twelve variables. With the latter approach, it was possible to identify the underlying relationships between the twelve questions.

**Figure 1:**
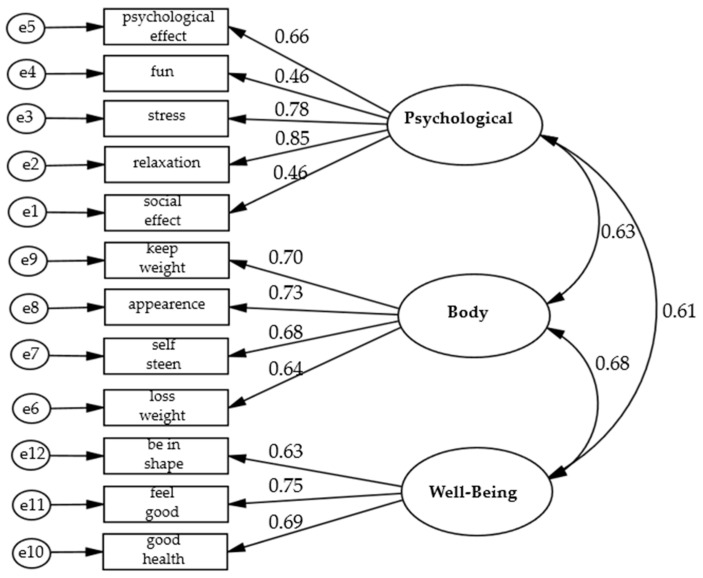
Factor analysis of questions about the motivations for practicing sport.

Figure 1 presents the results of the factor analysis, as well as the association coefficients between the twelve questions. Initially, an analysis of the principal components was carried out to verify the initial number of factors in the matrix. Afterward, three factors were fixed, and the Varimax rotation method with Kaiser normalization was used. Our analysis included the goodness-of-fit, using a variety of fit indices. A good model fit was determined when the chi-square ratio and the degrees of freedom (χ2/df) were < 3.0 [32], the Normed Fit Index (NFI) was > 0.80, the Comparative Fit Index (CFI) and Tucker–Lewis Index (TLI) were > 0.95 [33], and the Standard Root-Mean-Square Residue (SRMR) and Root-Mean-Square Error of Approximation (RMSEA) were < 0.08 [34]. The analysis indicated that the factorial model fits well (χ^2^(df) = 89.95 (32), (χ^2^/df) = 2.81, *p* < 0.001, NFI = 0.96, CFI = 0.95, TLI = 0.97, SRMR = 0.06, RMSEA = 0.06, 90% CI (0.44–0.66)). To calculate the convergent validity [32], factor loadings, Composite Reliability (CR), and Average Variance Extracted (AVE) were used, and the interpretations of the results were ≥ 0.70 and ≥ 0.50, respectively [35]. The analysis of the 12 items revealed 3 factors: (1) psychosocial—benefits of sport for mental health and social relationships, with factor loadings between 0.46 and 0.82 (CR = 0.902, AVE = 0.528) and relatively high internal consistency (Cronbach’s α = 0.778, McDonald’s ω = 0.761); (2) body—benefits of sport for physical health, with factor loadings from 0.57 to 0.80 (CR = 0.938, AVE = 0.552) and relatively high internal consistency (Cronbach’s α = 0.739, McDonald’s ω = 0.712); and (3) well-being—revealing the general benefits of sport for feeling good, with factor loadings from 0.67 to 0.79 (CR = 0.934, AVE = 0.543) and also relatively high internal consistency (Cronbach’s α = 0.782, McDonald’s ω = 0.774). All factors explained a total of 68% of the variance. The general reliability and internal consistency of the scale on the motivations for practicing sports indicated a Cronbach’s alpha of 0.885 and McDonald’s ω of 0.878, both rating as excellent [36].

### 2.3. Statistical Analysis

Initially, the Kolmogorov–Smirnov test was applied to assess whether the variables followed a normal distribution. Afterward, considering the normality of the data, continuous variables were presented as means and standard deviation (SD), while categorical data were presented as numbers (percentages). In the second step, analyses of variance (ANOVAs) were used to examine differences in the continuous variables between the three groups (i.e., adolescents, adults, and older adults). Therefore, intergroup differences were determined using Bonferroni’s post hoc tests. Considering that the occupation variable (categorical) was present in two of the three age groups, the between-group comparisons were calculated using a chi-square test. An unpaired Student’s parametric t-test processed statistical differences for the occupation variable. In the third step, Pearson’s correlation coefficients (*r*) were used to evaluate the relationship between the main variables of the study, considering the following interpretation: 0.1 = small, 0.3 = medium, and ≥0.5 = large [37]. In the fourth step, to test the effects of the three motivating factors (i.e., psychosocial, body, and well-being) on the results of PA-total (dependent variable) we conducted analyses of covariance (ANCOVAs). In separate models, we included a two-way interaction between (1) age groups and psychosocial motivational factor, between (2) age groups and body motivational factor, and between (3) age groups and well-being motivational factor. Moreover, between-group comparisons were processed using two models: the first unadjusted and the second controlled for the covariate sex. Subsequently, for each of the three motivational factors, intergroup effect sizes were calculated using partial eta squared [38], categorized as small (*η_p_*^2^ = 0.01), medium (*η_p_*^2^ = 0.06), and large (*η_p_*^2^ = 0.14). Finally, to deepen the understanding of the interaction effects of age x motivational factors, we performed exploratory analyses in terms of linear regressions separately in each of the three age groups. This procedure was performed only for significant interactions in the adjusted and unadjusted covariance models. For all tests, we considered a two-tailed *p*-value < 0.050 statistically significant. In the present study, factor analyses, goodness-of-fit, fit indices, and their graphical representations (Figure 1) were determined using the AMOS program. All other analyses were performed using IBM-SPSS (IBM Corp., Armonk, NY, USA) version 22.0.

## 3. Results

### Main Characteristics of the Sample

Among the study participants, 54.8% were women and 45.2% were men (*p* < 0.001) (see Table 1 for an overview). Overall, 23.5% were adolescents (14.7 years), 61.4% were adults (39.8 years), and 15.0% were older adults (68.0 years) (*p* < 0.001). Regarding the level of education, 96.6% of the adolescent group indicated the 3rd cycle, 37.6% of the adult group had a bachelor’s degree, and 70.7% of the older adult group stated a level of education up to the 3rd cycle (*p* < 0.001). Adolescents reported being mostly students (100.0%). Among adults, being working prevailed (73.9%); older adults revealed the occupation of retired (64.0%) (*p* < 0.001). Regarding the level of PA-total, adolescents had a higher score, followed by adults and older adults (*p* < 0.001). When asked about their motivations for practicing sports, adult individuals scored higher on the motivation-total scale (*p* < 0.001). The detailed analysis of this scale showed that, comparatively, adults showed greater motivation than adolescents and older adults to practice sports due to psychosocial factors, aspects related to the body, and interest in well-being (*p* < 0.001). In turn, older adults showed lower motivation than adolescents to participate in sports activities due to factors related to the body and well-being (*p* < 0.001).

Table 2 presents the correlation analysis results between the study’s main variables. Age indicated a significant positive and medium correlation with sex (*r* = 0.358). On the other hand, there were significant negative and medium associations between PA-total (*r* = -0.311) and well-being (*r* = −0.341). In turn, age also showed a significant positive and small association with the psychological (*r* = 0.264) and body (*r* = 0.288) motivational factors. Sex showed a significant positive and medium association with well-being (*r* = 0.318) and a positive and small significant association with psychosocial (*r* = 0.178) and body (*r* = 0.211) motivational factors. On the other hand, the association between sex and PA-total was significantly negative and small (*r* = −0.242). PA-total showed a significant positive and medium relationship with the psychological (*r* = 0.322) and body (*r* = 0.307) motivational factors in addition to a positive and small relationship with well-being (*r* = 0.294). The psychological factor indicated a positive and large association with the body factor (*r* = 0.540) and a positive and medium significant association with well-being (*r* = 0.470), while the body factor correlated positively with well-being (*r* = 0.539).

Table 3 presents the results of the univariate analysis. According to model I (unadjusted), there was a significant effect of the psychosocial motivational factor on PA-total [F(5.480)= 0.789, *p* = 0.017, *η_p_^2^* = 0.028]. On the other hand, there was no interaction effect for the interaction [F(10,480)= 0.945, *p* = 0.491, *η_p_^2^* = 0.019]. The body motivational factor showed no significant effect on PA-total [F(4.483)= 1.956, *p* = 0.100, *η_p_^2^* = 0.016, and there was also no significant effect for the interaction [F(8.483)= 0.654, *p* = 0.732, *η_p_^2^* = 0.011]. Regarding the well-being motivational factor, the analysis indicated a significant effect on PA-total [F(3.486)= 5.872, *p* = 0.001, *η_p_^2^* = 0.035], and a significant main effect was also revealed for the interaction [F(6.486)= 2.407, *p* = 0.027, *η_p_^2^* = 0.020].

The ANCOVA (model II) revealed that after controlling for sex, the psychological motivational factor remained significant [F(5.479)= 3.011, *p* = 0.011, *η_p_^2^* = 0.030], but, on the other hand, there was no significant interaction [F(10,479)= 0.898, *p* = 0.534, *η_p_^2^* = 0.018]. Regarding the body motivational factor, the analysis indicated a significant effect on total PA-total [F(4.482)= 2.589, *p* = 0.036, *η_p_^2^* = 0.034], while the interaction did not show a significant effect [F(8.482)= 0.534, *p* = 0.831, *η_p_^2^* = 0.009]. The well-being motivational factor remained with a significant effect on PA-total [F(3.485)= 5.775, *p* = 0.001, *η_p_^2^* = 0.034] and a significant main effect for the interaction [F(6.485)= 2.468, *p* = 0.028, *η_p_^2^*= 0.030].

The results of the linear regression analyses, specifying the effect of the motivational factor well-being on PA-total, separately for the three age groups, are presented in Table 4. Adolescents did not indicate significant results for the unadjusted model [OR = −0.033, *t* = −0.356, *p* = 0.722, R^2^ = 0.001], nor for the model adjusted by sex [OR = −0.017, *t* = −0.188, *p* = 0.851, R^2^ = 0.028]. Likewise, in the adult group, the unadjusted analysis showed no significant difference [OR = −0.058, *t* = 1.013, *p* = 0.312, R^2^ = 0.003], nor did the adjusted analysis [OR = −0.065, *t* = 1.145, *p* = 0.253, R^2^ = 0.031]. On the other hand, in the case of the older adult group, it was found that the motivational factor well-being showed a significant association in the unadjusted analysis [OR= 0.378, *t* = 3.485, *p* = 0.001, R^2^ = 0.243], which represented a 62% increase in the chance of improvement in the PA-total levels in this age group. Moreover, when controlled for sex, the analysis indicated a significant result [OR = 0.377, *t* = 3.444, *p* = 0.001, R^2^ = 0.288], representing an increase in the chance of PA-total by up to 63%.

## 4. Discussion

Our first objective was to report what motivates individuals in different age groups across the lifespan to be physically active. The investigation took place through a scale formed by twelve questions. The factor analysis suggested three motivating factors: psychological, body, and well-being. These factors represent a set of intrinsic and extrinsic motivations reported as a key element in an individual’s engagement in sports activities [29]. When asked about their motivations for practicing sports, adult individuals scored higher on the motivation-total scale. The detailed analysis of this scale showed that, comparatively, adults showed greater motivation than adolescents and older adults to practice sports due to psychosocial factors, aspects related to the body, and interest in well-being. In turn, older adults showed lower motivation than adolescents to participate in sports activities due to factors related to the body and well-being. Thus, this corroborates our first hypothesis, attesting that there were significant differences between psychological and well-being motivating factors according to age group. Both factors suggested the predominance of intrinsic motivation among respondents for sports practice, reflecting a personal interest in sports practice for pleasure and satisfaction [39].

Our second objective was to determine whether motivating factors influenced total PA levels and whether this differed across the lifespan. When controlling for sex, the three motivating factors (i.e., psychological, body, and well-being) showed a significant result on PA levels. On the other hand, the interaction with age group was significant only for the motivating factor of well-being. The findings not only attested to the participants’ desires to practice sports due to issues such as feeling good and having a satisfactory general state of health [10,40,41], but they also confirmed the differentiating role that the sex of individuals plays in exploring the behavioral factors that motivate sports practice throughout the lifespan [42,43].

An important finding of the present study was that the analysis of the effects of the age x motivational factors (exploratory analyses) interaction indicated a significant association of the motivating factor well-being with PA only in the older adult group. Specifically, the unadjusted analysis suggested that being 60 years old or older and being motivated to practice sports by the well-being factor represented a 62% chance of increasing levels of PA-total. Furthermore, when the analysis was adjusted for sex, the possibility of increasing PA-total levels increased up to 63%.

Based on these results, we partially confirmed our second hypothesis, attesting that older adults were motivated to practice sports due to the mental health benefits and not the association with the physical health benefits, as we had supposed. These findings do not align with previous investigations [11,12]. In these studies, the main motivator for practicing sports among older adults was the improvement of physical health. In the present study, it was observed that older adults did not devalue sports’ physical and functional benefits; however, they attributed greater value to psychological well-being, which was associated with good health and feeling good. Our findings are representative and important, as they increase the understanding of the reasons why different age groups adhere or do not adhere to sports [30]. Based on this information, it is possible for policymakers to effect changes in the behaviors of more specific community members according to age, sex, and/or interests [10]. In an investigation carried out in Malaysia [30], volunteers who regularly practiced PA with at least 150 min of moderate–vigorous physical activity (MVPA) during the last 6 months were analyzed in 2 groups: young adults (20 to 40 years; n = 763) and middle-aged adults aged 41 to 64 years (n = 597). According to the authors, men were more intrinsically motivated towards PA due to factors such as competition/ego, while women were more motivated towards PA due to the possibility of improving their appearance and physical condition. Our results corroborate the Malaysian outcomes according to the analysis of age groups. In this study, adolescents and younger adults were motivated to practice PA mainly due to extrinsic reasons, whereas with increasing age (i.e., middle-aged adults) the main motivating factor for PA was to benefit from psychological health.

In the present study, the analysis of intergroup differences (post hoc test) showed a clear contrast between the motivating factors of older adults to practice sports and those of adolescents and adults. We found that older adults were more motivated by intrinsic issues [43]. In contrast, adults and adolescents were more motivated by extrinsic issues related to the body and/or a certain appearance, which may be desired to meet the aesthetic standards demanded by society/others [44]. In proportional terms, adults indicated a higher level of PA-total than adolescents, and adolescents had a higher level than older adults. A point to highlight about adolescents and older adults is that, comparatively, adolescents have a personal desire to practice sports, as this practice is much more identified with their age group (i.e., fun and pleasure) [39]. Thus, unlike older adults (i.e., 80 years or older), adolescents see themselves as healthy, which facilitates/motivates them to maintain an active lifestyle through physical exercise [39]. When it comes to practicing sports, it is worth noting that regardless of whether the motivation is extrinsic or intrinsic, it must be voluntary. Presumably, when individuals feel pressured to exercise, they are likely to lack the pleasure and motivation to continue training, which can interrupt physically active behavior [45]. A possible explanation for the differences in views between older adults and adolescents/adults on the motivations for PA lies in the theory of socioemotional selectivity theory (SST) [46]. SST postulates that our motivations change as we age. Thus, it would be normal for individuals of different age groups to have different interests in exercising or even engaging in activities with moderate–high effort during the day. Therefore, among young people, motivations tend to be directed towards instrumental goals (future orientation), while older adults give importance to emotional goals (present orientation). According to Steltenpohl et al., [47], when it comes to PA, comparatively, adults have self-related motivations for exercise (me time), while older adults identify with exercise through social experience (we time).

There is no doubt that motivation is a determining factor for an individual to stay physically active [41,48]. A previous study with American university students (n = 98; 19.81 ± 2.38 years) revealed that extrinsic motivations for exercise determined worse psychological well-being, while intrinsic motivations led participants to better psychological well-being [43]. In another study conducted with 535 adolescents (14–18 years old) in America, the extrinsic motivating factor for practicing sports was prevalent [49]. Among females, the most commonly reported benefit was getting in shape, while men reported getting strong. An explanation for why young individuals tend to be motivated to practice sports by extrinsic factors is the inherent interest in approval or personal appreciation. This type of motivation is regulated in an introjected way, contrary to intrinsic motivation, which is characterized by the action of behavior aimed at pleasure and personal satisfaction [48]. It can be considered that the practice of sports for external reasons may occur to obtain other people’s approval or even due to some feeling of guilt, shame, low self-esteem, and/or low self-image [48].

In a population-based study that evaluated American adolescents (n = 1.661; 14.47 ± 1.61) to examine differences in PA motivation in three groups (i.e., PA at school, PA outside of school, and PA on the weekend) expressive results were verified [50]. At school, levels of moderate–vigorous physical activity (MVPA) were significantly associated with an external motivation (i.e., behavior to obtain reward) as well as with an internal motivation (i.e., search for autonomy and competence), an introjected motivation (i.e., relationship with feelings of guilt and pride), in addition to an identified motivation (i.e., engagement caused by valuing the behavior of another person). An out-of-school MVPA showed exactly the same types of motivational regulation as revealed for PA in school. In turn, on the weekend (leisure), MVPA levels were regulated by integrated and intrinsic motivation. The findings brought to light specificities about the modus operandi of adolescents’ motivations for PA according to the main places of their daily life. The findings are important, as they suggest the need for a differentiated plan capable of encouraging young people to practice exercise and make it a habit. 

### Limitations and Future Prospects

Our study has some limitations. First, the cross-sectional design does not allow for conclusions about the cause-and-effect relationship between PA and motivating factors for the practice of sport. Second, it is important to emphasize that the recruitment of participants in this investigation took place in different locations in the Funchal region. Therefore, it is possible that the PA levels of participants in the same age group differed. Third, there was a relative disproportion between the number of participants according to the three age groups. On the other hand, a strong point of this study was to bring to light comparative information about different age groups in a representative sample of people engaging in sports in the community. Fourth, the participants of this study were not physically evaluated. It is known that fitness function levels are directly associated with PA levels [3,51]; therefore, it is suggested that future studies include physical tests or batteries. Moreover, although PA levels were assessed using a validated questionnaire widely used in investigations [27], it is suggested that further studies include sensors (i.e., accelerometer-based devices) to continuously monitor daily levels of PA [52,53]. Regarding other implications for future research, it is suggested to explore in more depth the understanding of lifespan development regarding an individual’s motivation for attaining and maintaining an adequate level of PA, as well as its interdependence with health, quality of life, and well-being. From this perspective, it would be interesting to explore the relationship between previous experiences with a sport and the current motivation to promote and maintain PA levels. Another point to investigate is the relationships between PA and sociodemographic factors (i.e., sex, years of education, and own or family monthly income). In this case, mediation analyses could identify the associations between these factors. Moreover, quantitative designs can be useful for exploring the interpersonal and community levels. In contrast, a qualitative design enables a better understanding of motivating factors at the interpersonal level [10]. Finally, it is also suggested that longitudinal investigations be carried out to better understand changes throughout the lifespan regarding the motivation for PA, as well as its causes. In turn, understanding how motivators change with age can help keep individuals physically active and strengthen the maintenance of PA levels in different populations across the lifespan.

## 5. Conclusions

The presented findings provided important information about the behavior of the motivating factors for the practice of sport and, consequently, the promotion of PA levels in adolescents, adults, and older adults residing in the Autonomous Region of Madeira, Funchal, Portugal. The analysis revealed two intrinsic factors (i.e., psychosocial and well-being) and an extrinsic factor (i.e., body) as significant motivators common to the three age groups. After controlling for sex, we found that only the well-being factor showed a significant interaction with age. Therefore, by deepening the understanding of the effects of the age x motivational factors interaction, we found that the well-being factor was significant only among those aged 60 years or older. The present study brought to light information centered on the underlying reasons for the behavioral involvement of individuals in different age groups with exercise, which is fundamental to the promotion of PA levels. We conclude that from a lifespan perspective, it is essential to understand how individuals respond to stimuli. Thus, a possible strategy to motivate the practice of exercise is to first understand the specific interests of each sex and age group, and from that, plan actions according to the particularities of each population.

## Figures and Tables

**Table 1:** Main characteristics of the sample.

Variable	Overall (*n* = 498)	Adolescents (*n* = 117)	Adults (*n* = 306)	Older Adults (*n* = 75)	*p*-Value
Age (years)	38.16 ± 18.71	14.72 ± 1.25 ^b,c^	39.82 ± 11.30 ^c^	68.00 ± 6.64	<0.001
Sex (%)					<0.001
Men	225 (45.2)	77 (65.8) ^b,c^	137 (44.8) ^c^	11 (14.7)	
Women	273 (54.8)	40 (34.2)	169 (55.2)	64 (85.3)	
Education level (%)					<0.001
3rd cycle	253 (50.8)	113 (96.6) ^b,c^	87 (28.4)^c^	53 (70.7)	
Secondary level	121 (24.3)	4 (3.4) ^b,c^	104 (34.0)^c^	13 (17.3)	
Bachelor’s degree	123 (24.7)	-----	115 (37.6)^c^	8 (10.7)	
Occupation (%)					<0.001 ^†^
Student	152 (30.5)	117 (100.0)	35 (11.4)	-----	
Worker	238 (47.8)	-----	226 (73.9)	12 (16.0)	
Housewife	6 (1.2)	-----	3 (1.0)	3 (4.0)	
Unemployed	45 (9.0)	-----	38 (12.4)	7 (9.3)	
Retired	49 (9.8)	-----	1 (0.3) ^c^	48 (64.0)	
Invalid	8 (1.6)	-----	3 (1.0)	5 (6.7)	
Physical activity (n)					
PA-total	8.64 ± 1.31	8.91 ± 1.18 ^c^	8.65 ± 1.36	8.16 ± 1.60	<0.001
Motivational factors (n)					
Psychosocial	9.14 ± 1.40	8.90 ± 1.50 ^b^	9.27 ± 1.21	8.93 ± 1.58	0.014
Body	6.88 ± 1.38	6.66 ± 1.35 ^b^	7.03 ± 1.34 ^c^	6.57 ± 1.48 ^b^	0.006
Well-being	5.77 ± 0.61	5.78 ± 0.66	5.80 ± 0.58	5.62 ± 0.67	0.073
Motivation-total	21.79 ± 2.79	21.35 ± 2.94 ^b^	22.11 ± 2.58 ^c^	21.13 ± 3.19 ^b^	0.004

^b^*p* < 0.050 considering significant difference with adults; ^c^ *p* < 0.050 considering significant difference with older adults; and ^†^ *p* < 0.050 for Student’s t-test for two independent samples.

**Table 2:** Correlations between the key variables of the study.

Variables	1	2	3	4	5
1. Age	-----				
2. Sex (0= men, 1= woman)	0.358 **	-----			
3. PA-total	−0.311 **	−0.242 *	-----		
4. Psychosocial	0.264 *	0.178 *	0.322 *	-----	
5. Body	0.288 *	0.211 *	0.307 *	0.540 **	-----
6. Well-being	−0.341 *	0.318 *	0.294 *	0.470 **	0.539 **

PA-total = total physical activity score. * *p* < 0.010; ** *p* < 0.001.

**Table 3:** Results of univariate ANOVA analysis (unadjusted) and ANCOVA analysis (adjusted).

Variables	Adolescents (*n* = 117)	Adults (*n* = 306)	Older Adults (*n* = 75)	*p*-Value	Interaction Group Motivator	*η_p_* ^2^
Model I						
Psychological	9.77 ^b^	8.42 ^c^	8.07	0.017	0.491	0.019
Body	8.81	8.61	8.07	0.100	0.732	0.011
Well-being	9.40 ^c^	8.59 ^c^	7.50	0.001	0.027	0.030
Model II						
Psychological	8.67 ^c^	8.40 ^c^	8.17	0.011	0.534	0.018
Body	8.71 ^c^	8.56	8.21	0.036	0.831	0.009
Well-being	9.35 ^b^	8.56 ^c^	7.64	0.001	0.023	0.030

^b^*p* < 0.050 considering significant difference with adults; ^c^ *p* < 0.050 considering significant difference with older adults.

**Table 4:** Results of linear regression analyses for PA-total and well-being motivational factor.

	Adolescents OR 95% CI *p*-Value	Adults OR 95% CI *p*-Value	Older Adults OR 95% CI *p*-Value
Model I			
Well-being	−0.033 (−0.386–0.268) 0.722	−0.058 (−0.129–0.408) 0.312	0.378 (0.279–1.023) 0.001
R^2^	0.001	0.003	0.243
Model II			
Well-being	−0.017 (−0.356–0.295) 0.851	−0.065 (−0110–0.415) 0.253	0.377(0.273–1.025) 0.001
R^2^	0.028	0.031	0.288

R^2^ = R squared; OR= odds ratio. Model I = unadjusted model; model II = model adjusted by sex.

## Data Availability

The data presented in this study are available upon request from the corresponding author.
